# Correlating Cleaning Thoroughness with Effectiveness and Briefly Intervening to Affect Cleaning Outcomes: How Clean Is Cleaned?

**DOI:** 10.1371/journal.pone.0155779

**Published:** 2016-05-19

**Authors:** Robert Clifford, Michael Sparks, Eve Hosford, Ana Ong, Douglas Richesson, Susan Fraser, Yoon Kwak, Sonia Miller, Michael Julius, Patrick McGann, Emil Lesho

**Affiliations:** 1 Multidrug-resistant organism Repository and Surveillance Network, Walter Reed Army Institute of Research, Silver Spring, Maryland, United States of America; 2 Department of Infectious Diseases and Infection Control, Fort Belvoir Community Hospital, Fort Belvoir, Virginia, United States of America; Agricultural University of Athens, GREECE

## Abstract

**Objectives:**

The most efficient approach to monitoring and improving cleaning outcomes remains unresolved. We sought to extend the findings of a previous study by determining whether cleaning thoroughness (dye removal) correlates with cleaning efficacy (absence of molecular or cultivable biomaterial) and whether one brief educational intervention improves cleaning outcomes.

**Design:**

Before-after trial.

**Setting:**

Newly built community hospital.

**Intervention:**

90 minute training refresher with surface-specific performance results.

**Methods:**

Dye removal, measured by fluorescence, and biomaterial removal and acquisition, measured with culture and culture-independent PCR-based assays, were clandestinely assessed for eight consecutive months. At this midpoint, results were presented to the cleaning staff (intervention) and assessments continued for another eight consecutive months.

**Results:**

1273 surfaces were sampled before and after terminal room cleaning. In the short-term, dye removal increased from 40.3% to 50.0% (not significant). For the entire study period, dye removal also improved but not significantly. After the intervention, the number of rooms testing positive for specific pathogenic species by culturing decreased from 55.6% to 36.6% (not significant), and those testing positive by PCR fell from 80.6% to 53.7% (P = 0.016). For nonspecific biomaterial on surfaces: a) removal of cultivable Gram-negatives (GN) trended toward improvement (P = 0.056); b) removal of any cultivable growth was unchanged but acquisition (detection of biomaterial on post-cleaned surfaces that were contaminant-free before cleaning) worsened (P = 0.017); c) removal of PCR-based detection of bacterial DNA improved (P = 0.046), but acquisition worsened (P = 0.003); d) cleaning thoroughness and efficacy were not correlated.

**Conclusion:**

At this facility, a minor intervention or minimally more aggressive cleaning may reduce pathogen-specific contamination, but not without unintended consequences.

## Introduction

Eliminating healthcare-associated infections (HAI) is a public health priority in the United States [1], and increasingly attracts the interest of payers, legislators, regulators, consumer groups and the general public [2]. Accumulating evidence implicates environmental contamination in HAI transmission [3–5], with adequate cleaning of environmental surfaces a vital HAI prevention strategy [6]. Additionally, as the pipeline of new antibiotics remains dry and we lose treatment options to increasing antibiotic resistance, effective cleaning becomes more crucial. Although leading experts continue to debate the optimal approach for assessing relationships between biomaterial and cleaning outcomes, they agree that more sensitive detection assays are needed [4, 7], along with comparative effectiveness assessments and linking study results to patient centered outcomes [8].

An underestimated driver of antimicrobial resistance and transmissibility of nosocomial pathogens is the presence of DNA on hospital surfaces [9, 10]. Previously considered clinically inconsequential, this material may be present in viable (and perhaps uncultivable) bacteria, in dead cells, or even in extracellular form available for uptake by competent cells. Recent observations that pathogenic bacteria can integrate even short, damaged DNA fragments into their chromosomes expand the potential implications of this contaminant type [9]. Indeed, we recently reported that levels of environmental *E*. *coli* DNA were correlated with the number of inpatient *E*. *coli* infections (P < 0.005) [10]. Together, these findings highlight need for surveillance efforts to utilize not only culture-based methods, but also culture-free methods such as PCR.

The adequacy and efficiency of measures used to assess cleaning staff performance also merits careful attention [4, 7, 8, 11]. The most efficient approach to monitor and improve cleaning outcomes and whether cleaning thoroughness correlates with DNA removal remain important unanswered questions. Here, we define cleaning *thoroughness* as whether 90% of the invisible marking dye DAZO® has been removed [10, 12] and cleaning *efficacy* as whether a surface has detectable biomaterial following terminal cleaning. Biomaterial is further separated into species-specific or total nonspecific, and detected either by PCR or culture, and ‘efficacy’ includes both removal of biomaterial from previously dirty surfaces, and absence of biomaterial on post-cleaned surfaces that were contaminant-free before cleaning.

Conventional paradigms and intuition suggest that the more training or education and follow-up ‘refresher’ sessions the better. However, it has been shown that a single, brief intervention session may favorably affect behavioral outcomes, such as when a physician mentions the importance of smoking cessation or weight loss in a single patient encounter [13–15]. It has also been shown that repeated and intense interventions can impact cleaning outcomes [16, 17]; less explored is whether a single, brief intervention session can improve cleaning staff performance. If effective, one brief educational session would be preferable to more numerous long sessions for several reasons. First, a single session would require less time and money, and would minimize the “training fatigue” associated with a hospital staff obligated to complete an ever-increasing number of training requirements. Second, in some hospitals for several reasons (extended absences, short term hires, competing demands, etc,) personnel might not have the opportunity to receive multiple reinforcing sessions. Finally, interventions have not been widely studied at evidence-based design (EBD)-facilities. Although a single, brief intervention might be ineffective at a conventional facility, in an EBD hospital, with the panoply of patient safety, staff ergonomic, and environmental features [18], it may be sufficient to see an effect.

We sought to extend the finding of a previous study by determining whether one brief intervention could improve the performance of cleaning staff at a newly constructed evidence-based design Military Healthcare System facility, the 120-bed Fort Belvoir Community Hospital in northeastern Virginia, for both short- and longer-term durations, using a previously collected data set [10]. Furthermore, we stratified performance outcomes into: a) the removal of biomaterial from surfaces that were dirty before cleaning, and b) not contaminating post-cleaned surfaces that were free of biomaterial before cleaning (acquisition). Last, we sought to determine whether thorough cleaning was associated with effective cleaning, using cultures in tandem with a novel molecular approach recently published [10].

## Methods

The study was undertaken as a quality improvement—infection control project authorized as exempted non-human research by Walter Reed Army Institute of Research Institutional Review Board NHSR protocol number 1761. Surveillance at the FBCH was prospectively conducted with 18 surveillance events occurring over a 16-month period (October, 2011 to February, 2013). During the first nine months sampling was clandestinely conducted and the cleaning staff was not aware performance was being measured. A single feedback and training refresher intervention event occurred in June 2012. The intervention consisted of a ninety minute educational session during which results of the previous nine months of monitoring cleaning thoroughness at the ward, room, and individual surface levels were provided to cleaning and infection control staff. A pre-validated script was also followed using guidelines made available by the Centers for Disease Control and Prevention [12]. During the study period no changes in average daily census, cleaning procedures, protocols or staff occurred. There were no outbreaks, and this study was not conducted in response to an outbreak.

Detailed methods for room sampling, culture, and PCR-based assays were described previously [10]. Briefly, following patient discharge, but prior to terminal cleaning, 17-high touch surfaces were sampled for 20 seconds using a rayon-tipped swab pre-moistened with nutrient transport media. These surfaces were then marked with an invisible liquid dye (DAZO®). After terminal cleaning, the presence or absence of dye was assessed with ultraviolet light and recorded using a hand-held device, the Encompass monitoring system (EcoLab, St. Paul, MN). Surfaces were then resampled as described above. Cleaning of a surface was deemed to be thorough if at least 90% of the marking dye was removed. To assess cleaning efficacy using culture-based methods, swabs were streaked onto blood (“BAP”) and MacConkey (“MAC”) agar plates. Bacteria that grew on BAP after 24–48 hours of incubation at 35°C were taxonomically identified using the Phoenix automated system (Bekton Dickenson, Franklin Lakes, NJ) with the PMIC/ID-107 panel. Culture-based target organisms (“TO”) in this study were *Acinetobacter baumannii/ Acinetobacter baumannii calcoaceticus* complex (Acb); *Escherichia coli* (Eco); *Pseudomonas aeruginosa* (Psa); *Staphylococcus aureus* (Staph); *Klebsiella pneumoniae* (Kpn); *Enterococcus fecium/ faecalis* (Enteroc); and *Enterobacter cloaceae/ aerogenes* (Enterob).

To assess efficacy with a molecular approach two PCR assays were used: a) a general 16S rDNA PCR (“16S”) that detects the presence of 94% of all known bacteria with high sensitivity (1 x 10^2^ copies of purified genomic DNA) [19]; and b) a set of species-specific PCR assays that detect *Clostridium difficile*, Eco, Psa, Staph, Kpn, and Acb [10].

We examined four conditions: i) the effect of the intervention on dye removal; ii) the effect of the intervention on the presence of pathogenic bacterial species at the whole-room level; iii) the effect of the intervention on biomaterial removal and accumulation on high-touch surfaces; and iv) whether dye removal correlated with biomaterial removal on surfaces. Removal was defined as no culture or PCR-based detection of biomaterial on post-cleaned surfaces that were dirty / contaminated before cleaning. Acquisition was defined as detection of biomaterial on post-cleaned surfaces that were clean / contaminant-free before cleaning. Fisher’s exact test was used to assess statistical significance. To mitigate sampling biases by allowing for naturally occurring, and/or worker-driven re-contamination processes to occur, both the same area and also adjacent/ surrounding areas were sampled during the post cleaning sampling.

Role of the Funding Source: The U.S. Army Medical Command and the Department of Defense Global Emerging Infection Surveillance and Response System had no role in the study design, data collection, data analysis, or manuscript preparation.

## Results

1273 surfaces from 78 rooms were sampled before and after terminal room cleaning (yielding 2546 swabs). Eight surveillance visits to 36 rooms occurred before the intervention and 10 visits to 41 rooms afterwards. 50 percent of the rooms were sampled both before and after the intervention.

### Effect of Intervention on Surface-Level Cleaning Thoroughness

We observed differences in cleaning thoroughness rates for individual surfaces (Table 1). The call box exhibited the greatest improvement in cleaning thoroughness (19 percentage points) after the intervention and the room chair had the largest decrease in thoroughness (39 percentage points). Overall, DAZO-measured cleaning thoroughness was unchanged (0.02 percentage point increase) following the intervention (Table 1). For the more limited observation period (two months immediately before and after the intervention) overall cleaning thoroughness increased 9.68 percentage points, suggesting that the intervention had a short-term beneficial effect. Cleaning thoroughness improved most dramatically for the bedside table in the shorter term immediate pre and post intervention period (+57.58 percentage points); this amount was matched by the largest observed decrease in performance (room chair, -57.58 percentage points). 11 of the 17 surface types showed an increase of 5 or more percentage points in the two-month period following the intervention, (P = 0.06) while 4 surfaces showed a decrease in cleaning thoroughness during this time period (see Table 1).

**Table 1:** Cleaning Thoroughness (Dye Removal).

10-13-11–1-17-13 (all data)									
	Pre-intervention			Post-intervention			Comparative Results		
Surface Type	Pass	Fail	Pass Rate	Pass	Fail	Pass Rate	Improved?	Pass Rate Change	P-value
room chair	24	9	72.73%	14	27	34.15%	FALSE	-38.58%	0.0012
tray table	33	3	91.67%	32	8	80.00%	FALSE	-11.67%	0.1986
bathroom lightswitch	9	27	25.00%	7	34	17.07%	FALSE	-7.93%	0.4148
room lightswitch	5	28	15.15%	3	38	7.32%	FALSE	-7.83%	0.4538
bedside table	18	17	51.43%	18	23	43.90%	FALSE	-7.53%	0.6454
toilet seat	32	3	91.43%	36	5	87.80%	FALSE	-3.62%	0.7188
bedpan cleaner	13	22	37.14%	14	27	34.15%	FALSE	-3.00%	0.8141
side rail	10	24	29.41%	11	28	28.21%	FALSE	-1.21%	1.0000
IV pole	17	12	58.62%	18	13	58.06%	FALSE	-0.56%	1.0000
bathroom door closer	19	17	52.78%	23	18	56.10%	TRUE	3.32%	0.8212
toilet handle	8	27	22.86%	12	29	29.27%	TRUE	6.41%	0.6065
room sink	9	24	27.27%	14	27	34.15%	TRUE	6.87%	0.6170
toilet rail	4	32	11.11%	9	32	21.95%	TRUE	10.84%	0.2380
bathroom sink	15	21	41.67%	22	19	53.66%	TRUE	11.99%	0.3627
room door closer	6	30	16.67%	12	29	29.27%	TRUE	12.60%	0.2810
telephone	17	17	50.00%	26	15	63.41%	TRUE	13.41%	0.3484
call box	11	24	31.43%	20	20	50.00%	TRUE	18.57%	0.1581
Total	250	337	42.59%	291	392	42.61%	TRUE	0.02%	1.0000
**4-19-12–7-24-12 (subset)**									
room chair	10	1	90.91%	4	8	33.33%	FALSE	-57.58%	0.0094
tray table	10	1	90.91%	8	3	72.73%	FALSE	-18.18%	0.5865
bathroom lightswitch	3	8	27.27%	2	10	16.67%	FALSE	-10.61%	0.6404
IV pole	5	5	50.00%	4	5	44.44%	FALSE	-5.56%	1.0000
bathroom sink	6	5	54.55%	6	6	50.00%	FALSE	-4.55%	1.0000
room lightswitch	1	10	9.09%	1	11	8.33%	FALSE	-0.76%	1.0000
room sink	4	7	36.36%	5	7	41.67%	TRUE	5.30%	1.0000
toilet handle	4	7	36.36%	5	7	41.67%	TRUE	5.30%	1.0000
toilet seat	10	1	90.91%	12	0	100.00%	TRUE	9.09%	0.4783
bedpan cleaner	4	7	36.36%	6	6	50.00%	TRUE	13.64%	0.6802
side rail	2	9	18.18%	4	7	36.36%	TRUE	18.18%	0.6351
bathroom door closer	6	5	54.55%	9	3	75.00%	TRUE	20.45%	0.4003
toilet rail	1	10	9.09%	4	8	33.33%	TRUE	24.24%	0.3168
room door closer	1	10	9.09%	4	8	33.33%	TRUE	24.24%	0.3168
call box	3	8	27.27%	6	5	54.55%	TRUE	27.27%	0.3870
telephone	4	7	36.36%	11	1	91.67%	TRUE	55.30%	0.0094
bedside table	1	10	9.09%	8	4	66.67%	TRUE	57.58%	0.0094
Total	75	111	40.32%	99	99	50.00%	TRUE	9.68%	0.0650

Effects of intervention on cleaning thoroughness. Pass: > = 90% of the DAZO marker was removed during cleaning. Fail: < 90% of DAZO was removed. Surfaces are ordered by the change in the rate of DAZO removal seen after the training intervention. P-values are for a two-tailed Fisher’s exact test. Counts from the full observation period are shown, as well as those from a reduced observation period spanning two months before and after the intervention.

### Effect of Intervention on Ward-Level Detection of Species Specific Target Organisms

**Fig 1:**
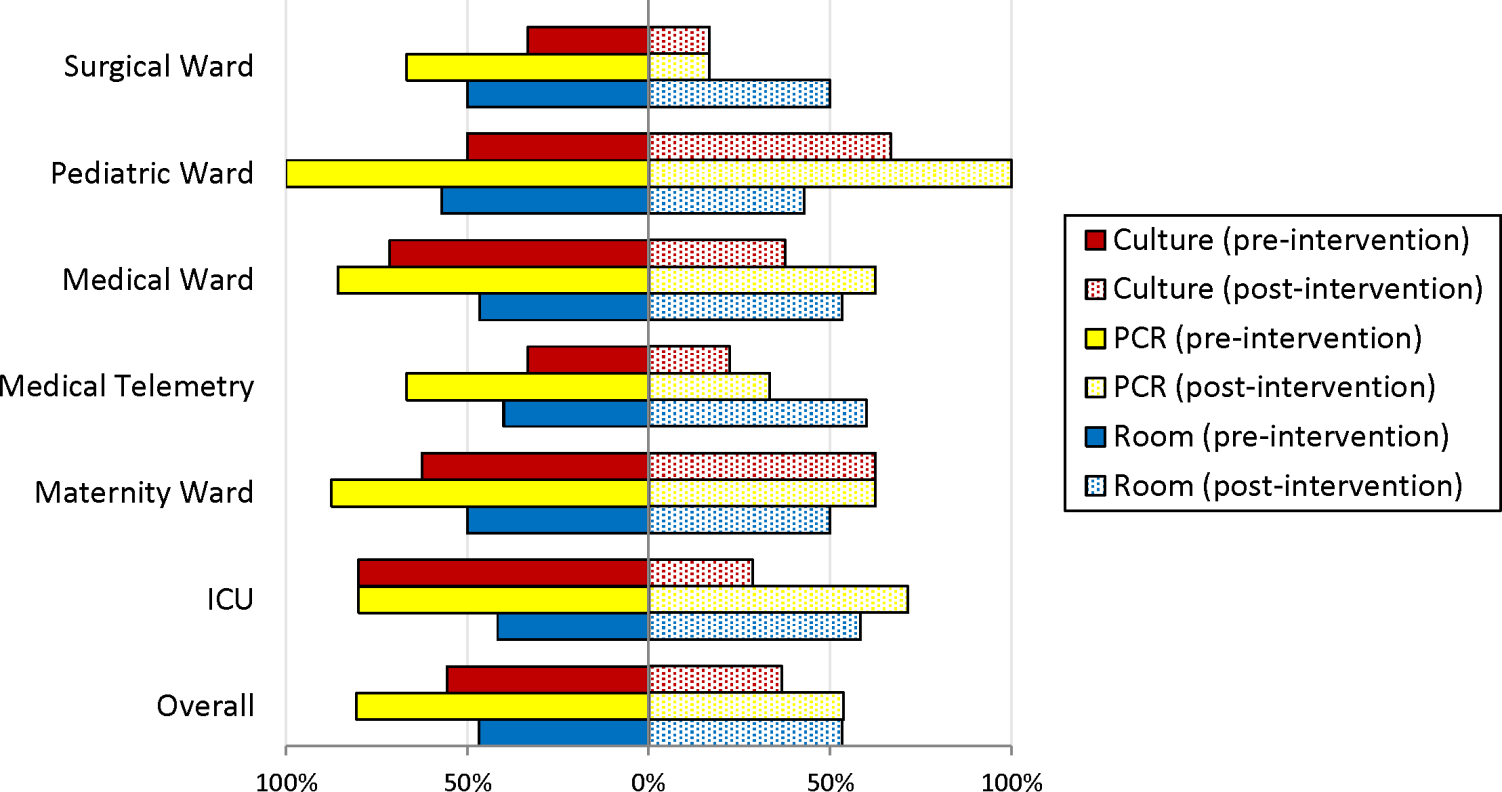
Pathogen Presence by Hospital Unit Type. Percentage of rooms testing positive for any of seven target organisms associated with hospital-acquired infections by culturing (red bars) or PCR (yellow bars) before and after the cleaning intervention. Blue bars show the percentage of rooms assayed before the intervention and after the intervention.

Because target organisms, namely species frequently associated with hospital acquired infections, were rarely encountered, we examined whether the frequency at which they were detected on any surface in a room changed after the cleaning intervention. In each hospital unit type, target organisms were detected at least as frequently with 16S PCR as with BAP-based culturing (Fig 1 solid bars). Following the intervention, the ICU exhibited the greatest reduction in the percentage of rooms in which TO were detected by BAP (16.7 percentage point difference (Fig 1, stippled bars), and the surgical ward showed the greatest reduction in the number of rooms testing positive for TO by 16S (25.0 points fewer). Interestingly, a greater percentage (8.33 points more) of ICU rooms tested positive for TOs by 16S PCR following the intervention, and was the only unit seen to fare worse following the event using either detection measure. As measured by BAP, no change in the percentage of rooms containing TO was observed for the pediatric ward, medical telemetry, and maternity ward after intervention.

### Effect of Intervention on Surface-Level Detection of Nonspecific Biomaterial

Culture-based and PCR-based results were similar for all surfaces in aggregate (Table 2). Detection of biomaterial by MAC culturing, which measures the presence of viable Gram-negative bacteria, was lower than the other two methods.

**Table 2:** Biomaterial removal and acquisition before and after the training intervention.

**A) Removal of Biomaterial**							
	**Before intervention**			**After Intervention**			
**Assay**	**Pos/Neg**^**1**^	**Pos/Pos**^**2**^	**% Remove**	**Pos/Neg**	**Pos/Pos**	**% Remove**	**P** =
16S	124	163	43.21	152	142	51.7	***0*.*046***
BAP	156	168	48.15	186	193	49.08	0.821
MAC	57	32	64.04	82	24	77.36	*0*.*056*
**B) Acquisition of Biomaterial**							
	**Before intervention**			**After Intervention**			
**Assay**	**Neg/Neg**^**3**^	**Neg/Pos**^**4**^	**% Acquire**	**Neg/Neg**	**Neg/Pos**	**% Acquire**	**P** =
16S	240	63	20.79	269	120	30.85	***0*.*003***
BAP	216	50	18.8	221	83	27.3	***0*.*017***
MAC	455	46	9.18	536	41	7.11	0.219

1 Tested positive for biomaterial before terminal cleaning, negative after terminal cleaning

2 Tested positive for biomaterial before terminal cleaning, positive after terminal cleaning

3 Tested negative for biomaterial before terminal cleaning, negative after terminal cleaning

4 Tested negative for biomaterial before terminal cleaning, negative after terminal cleaning

Detection of biomaterial on 1273 individual high-touch surfaces. Pos/Neg: the surface tested positive for biomaterial before terminal cleaning, and negative after terminal cleaning. Pos/Pos: tested positive for biomaterial before and after terminal cleaning. Neg/Neg: tested negative for biomaterial before and after terminal cleaning. Neg/Pos: tested negative for biomaterial before terminal cleaning, and positive after terminal cleaning. P-values are for a two-tailed Fisher’s exact test.

For all surfaces combined biomaterial removal increased as measured by every detection assay after the training intervention. Improved removal of bacterial DNA as measured by the 16S rDNA assay was significant (P = 0.046) and the increased removal of viable Gram-negative bacteria as measured by growth of MAC medium approached significance (P = 0.056) (Table 2A).

Unexpectedly, we observed increased acquisition of non-specific biomaterial on clean surfaces after the intervention. For all surfaces combined, acquisition of biomaterial increased significantly as measured by 16S rDNA detection (P = 0.003) and growth on BAP (P = 0.017). In contrast, the accumulation of viable Gram-negative bacteria on previously clean surfaces fell from 9.18% to 7.11%, although this change is not statistically significant (Table 2B, MAC assay).

#### Was Cleaning Thoroughness Correlated with Efficacy?

We examined whether the thoroughness of terminal room cleaning correlated with the removal of biomaterial from dirty surfaces. We found no significant association between DAZO removal and the detection of bacterial DNA (Table 3A). Likewise, we observed no association between cleaning thoroughness and by bacterial growth on BAP and MAC media. Thus cleaning thoroughness did not correlate with the removal of DNA or cultivable bacteria from contaminated surfaces.

**Table 3:** Relationship between DAZO Removal and Cleaning Outcome.

A) Removal of biomaterial					
	DAZO pass		DAZO fail		
Assay	Pos/Neg	Pos/Pos	Pos/Neg	Pos/Pos	*P* =
16S	131	118	174	155	1.0000
BAP	157	168	204	173	0.1304
MAC	28	64	28	75	0.6374
B) Acquisition of biomaterial					
	DAZO pass		DAZO fail		
Assay	Neg/Neg	Neg/Pos	Neg/Neg	Neg/Pos	*P* =
16S	205	87	304	96	0.0974
BAP	155	61	281	71	**0.0316**
MAC	411	38	577	49	0.7344

Detection of biomaterial on 541 surfaces that were thoroughly cleaned (DAZO pass) and 729 surfaces that were not thoroughly cleaned (DAZO fail). Pos/Pos, Pos/Neg, Neg/Neg and Neg/Pos are as described in Table 2. P-values are for a two-tailed Fisher’s exact test.

We also examined whether thoroughness of cleaning correlated with the accumulation of biomaterial on surfaces that were free of biomaterial before terminal cleaning (Table 3B). We saw no significant association between dye removal and the detection of bacterial DNA by PCR and Gram-negative growth on MAC medium. However, detection of microbes using BAP medium, which supports growth of both Gram-positive and Gram-negative bacteria, did occur more often on thoroughly cleaned surfaces that initially lacked biomaterial (P = 0.03) (Table 3B). A greater percent of these surfaces were found to have acquired biomaterial among DAZO-passed surfaces (28%) relative to those that failed (20%).

## Discussion

Nearly 40% of high-touch hospital surfaces determined to be thoroughly cleaned (based on a widely used monitoring system) with a quaternary ammonium disinfectant used according to established guidelines still harbored viable microbes and bacterial DNA. We previously reported that levels of environmental *E*. *coli* DNA correlated with the frequency of inpatient *E*. *coli* infections in this setting [10]. Following a single brief performance feedback and training session, cleaning thoroughness trended toward improvement. At the level of hospital unit, target pathogens were detected less frequently after the intervention. Further, the removal of DNA from individual surfaces that had biomaterial before terminal cleaning significantly increased and the removal of Gram-negative organisms showed improvement. However, the acquisition of all viable microbes (Gram-positive and Gram-negative) and bacterial DNA on post-cleaned surfaces that were free of viable organisms before cleaning significantly worsened. Our findings suggest that a brief cleaning intervention may increase cleaning efforts and reduce pathogen-specific biomaterial, but not without unintended consequences.

By linking results to HAI rates, and providing a comparative assessment of two widely used monitoring systems (fluorescent dye removal and cultures) to a sensitive and specific molecular assay, this study addresses some of the critical knowledge gaps recently listed in a systematic review of environmental cleaning [8]. By providing materials and concepts for enhanced monitoring and training of environmental cleaning, the findings readily translate into better practices and improved patient safety.

First, the review by Han and coworkers stated that an important remaining knowledge gap is incorporating patient-centered outcome data in cleaning studies. During the data acquisition period for this study, we determined that environmental levels of *E*. *coli* DNA were correlated with frequency of inpatient *E*.*coli* infections [10].

Our results suggest that the mere training of cleaning personnel without changing the cleaning methods is unlikely to result in significant improvement of hygienic quality of hospital surfaces. With that in mind, consideration should be given to revising educational and training materials for cleaning staff. The revision should include the importance of environmental DNA in HAI, the potential for more vigorous efforts to increase contamination, especially with DNA, and noting the increasing discoveries of disinfectant tolerant or resistant organisms [10, 21]. The revision could also re-emphasize the proper use of cleaning wipes and not passing more than one time with the same side of the wipe.

Second, Han *et al*.’s review also recommended comparative assessments of methods for monitoring cleaning. Here we provide results based on culture-dependent assays, culture-independent assays, pathogen-specific, and overall nonspecific biomaterial assays, and compare them to each other and to a current reference standard for thoroughness (DAZO). This study also validates the usefulness and highlights the value of the culture-independent, highly sensitive 16S rDNA general bacterial and the species–specific PCR assays we developed. The later can easily be modified to include primers for nearly any target pathogen of interest.

The significant reduction in molecular detection of target organisms frequently associated with nosocomial infections, along with trends in thoroughness and removal of general nonspecific biomaterial, suggest that intervention had some impact and that the cleaning staff was attempting to clean more vigorously. This is further supported by finding that cleaning thoroughness improved for four of the five surfaces most frequently harboring cultivable biomaterial, while it decreased for four of the five surfaces least likely to harbor the same (Table 1). This is consistent with the cleaning staff redirecting their efforts to the most poorly cleaned / dirtiest surfaces at the expense of the least contaminated surfaces after receiving the surface-specific results during the intervention (Table 1 and S1 Table). Notably, acquisition significantly worsened after the intervention (Table 2).

Our findings suggest that even a minimal intervention with good intention can have untoward effects. Perhaps performance feedback at the surface-specific level is a double edged sword and fosters the natural human tendency to take shortcuts or pay less attention to areas believed to be trouble free. Or, as Rupp *et al*. found, time spent cleaning is not correlated with cleaning thoroughness [17]. Perhaps another revision to training materials for cleaning staff should be a reminder that if time is limited, reallocating cleaning efforts among surfaces can be counterproductive. In other words, it is not necessary to ‘rob Peter to pay Paul.’

While thorough cleaning does not guarantee effective cleaning, the successful removal of contaminants without additional deposition of biomaterial (especially DNA or biocide tolerant organisms) might indeed necessitate more time spent cleaning. An alarming possibility is that isolates might be adapting to disinfectants, creating biofilms, and multiplying in cleaning solutions. 47% of disinfection solutions were found to contain bacteria [20, 21]. Indeed, Manian, *et al*. found that *A*. *baumannii* and MRSA were frequently isolated after as many as four rounds of cleaning and disinfection [22]. Carling, *et al*. found a significantly (p = <0.0001) higher number of surfaces contained cultivable biomaterial when they were equivalently cleaned with the standard quaternary ammonium compound compared to peracetic acid/hydrogen peroxide compound [23].

Others have described a so-called “rebound effect,” in which a period of relaxed cleaning following intensified cleaning efforts results in a substantive increase in aerobic colony counts [24]. There also exists the possibility of a “retaliation effect,” in which continuous, vigorous cleaning can generate an elevated infection hazard by liberating otherwise-sequestered genetic material encoding virulence and/or resistance determinants, thereby making these available for uptake by competent cells [24–26]. The importance of preventing surface recontamination to guarantee a safe environment for patients through cleaning with agents capable of inhibiting microbial recontamination or using surfaces “resistant” to recontamination has been emphasized [5]. In an attempt to mitigate surface recontamination or the rebound effects, some have turned to probiotics, and have demonstrated a beneficial impact on the inanimate environmental resistome [27].

This study has several limitations. It was a single facility study that serves a predominantly military population. However, all the cleaning staff are civilians, and the hospital treats patients of all ages and races including newborns and elderly retirees. Strengths of this study are that it was conducted at community hospital (where the majority of healthcare delivery occurs in the U.S.) and it was not carried out not in response to an outbreak [8]. One might also argue that the intervention was too short and imparted just enough influence to create harm. However, our intent was to attempt to determine if a performance improvement intervention could retain effectiveness while maximizing its efficiency by reducing it to a single brief session.

Finally, we were unable to use direct application Rodac plates or films on the surfaces. Therefore, streaking the swabs on plates likely lowered the percentage of detectable cultivable microorganisms.

Our findings suggest that, at this facility, a brief cleaning intervention may increase cleaning efforts and reduce pathogen-specific biomaterial, but not without unintended consequences. One possible way forward is to see if either a single longer educational intervention, or two brief sessions, with the above concepts included (ideally at multiple centers), produces better outcomes and less contaminant acquisition.

## Disclaimer

Material has been reviewed by the Walter Reed Army Institute of Research. There is no objection to its presentation and/or publication. The opinions or assertions contained herein are the private views of the author, and are not to be construed as official, or as reflecting true views of the Department of the Army or the Department of Defense. There are no conflicts of interest.

## Supporting Information

S1 Table Contamination Frequency of Specific Surfaces Before & After the Intervention. Surfaces are ordered by the change in the contamination frequency after the training intervention. P-values are for a two-tailed Fisher’s exact test. Counts from the full observation period are shown.
(DOCX)
