# Supplementary material for: Correlating Cleaning Thoroughness with Effectiveness and Briefly Intervening to Affect Cleaning Outcomes: How Clean Is Cleaned?
Source: PLoS One. 2016 May 19;11(5):e0155779. doi: 10.1371/journal.pone.0155779 (PMC4873012; doi:10.1371/journal.pone.0155779)
Supplement: S1 Table — Surfaces are ordered by the change in the contamination frequency after the training intervention. P-values are for a two-tailed Fisher’s exact test. Counts from the full observation period are shown. (DOCX) [file pone.0155779.s001.docx]

| **S1 Table: Contamination Frequency of Specific Surfaces Before and After the Intervention** | | | | | | | | | | |
| --- | --- | --- | --- | --- | --- | --- | --- | --- | --- | --- |
|  |  | Pre-intervention | | | Post-intervention | | | Comparative Results | | |
|  | Surface Type | Pass | Fail | Pass Rate | Pass | Fail | Pass Rate | Improved? | Pass Rate Differential | P-value |
|  |  |  |  |  |  |  |  |  |  |  |
| 16S rDNA | toilet handle | 10 | 1 | 90.91% | 6 | 6 | 50.00% | FALSE | -40.91% | 0.0686 |
|  | room lightswitch | 10 | 1 | 90.91% | 7 | 5 | 58.33% | FALSE | -32.58% | 0.1550 |
|  | toilet rail | 8 | 3 | 72.73% | 6 | 6 | 50.00% | FALSE | -22.73% | 0.4003 |
|  | tray table | 8 | 3 | 72.73% | 6 | 5 | 54.55% | FALSE | -18.18% | 0.6594 |
|  | room door closer | 10 | 1 | 90.91% | 10 | 2 | 83.33% | FALSE | -7.58% | 1.0000 |
|  | bedpan cleaner | 9 | 2 | 81.82% | 9 | 3 | 75.00% | FALSE | -6.82% | 1.0000 |
|  | call box | 9 | 2 | 81.82% | 9 | 2 | 81.82% | FALSE | 0.00% | 1.0000 |
|  | toilet seat | 9 | 2 | 81.82% | 10 | 2 | 83.33% | TRUE | 1.52% | 1.0000 |
|  | telephone | 8 | 3 | 72.73% | 9 | 3 | 75.00% | TRUE | 2.27% | 1.0000 |
|  | IV pole | 7 | 3 | 70.00% | 7 | 2 | 77.78% | TRUE | 7.78% | 1.0000 |
|  | side rail | 9 | 2 | 81.82% | 10 | 1 | 90.91% | TRUE | 9.09% | 1.0000 |
|  | bedside table | 8 | 3 | 72.73% | 10 | 2 | 83.33% | TRUE | 10.61% | 0.6404 |
|  | bathroom door closer | 7 | 4 | 63.64% | 9 | 3 | 75.00% | TRUE | 11.36% | 0.6668 |
|  | bathroom lightswitch | 7 | 4 | 63.64% | 9 | 3 | 75.00% | TRUE | 11.36% | 0.6668 |
|  | room sink | 6 | 5 | 54.55% | 8 | 4 | 66.67% | TRUE | 12.12% | 0.6802 |
|  | bathroom sink | 6 | 5 | 54.55% | 8 | 4 | 66.67% | TRUE | 12.12% | 0.6802 |
|  | room chair | 6 | 5 | 54.55% | 8 | 4 | 66.67% | TRUE | 12.12% | 0.6802 |
|  | Total | 137 | 49 | 73.66% | 141 | 57 | 71.21% | FALSE | -2.44% | 0.6481 |
|  |  |  |  |  |  |  |  |  |  |  |
| Blood Agar | tray table | 9 | 2 | 81.82% | 3 | 8 | 27.27% | FALSE | -54.55% | 0.0300 |
|  | toilet seat | 9 | 2 | 81.82% | 6 | 6 | 50.00% | FALSE | -31.82% | 0.1930 |
|  | telephone | 8 | 3 | 72.73% | 6 | 6 | 50.00% | FALSE | -22.73% | 0.4003 |
|  | toilet rail | 6 | 5 | 54.55% | 4 | 8 | 33.33% | FALSE | -21.21% | 0.4136 |
|  | bathroom sink | 6 | 5 | 54.55% | 5 | 7 | 41.67% | FALSE | -12.88% | 0.6843 |
|  | side rail | 11 | 0 | 100.00% | 10 | 1 | 90.91% | FALSE | -9.09% | 1.0000 |
|  | room door closer | 11 | 0 | 100.00% | 11 | 1 | 91.67% | FALSE | -8.33% | 1.0000 |
|  | room sink | 7 | 4 | 63.64% | 7 | 5 | 58.33% | FALSE | -5.30% | 1.0000 |
|  | bedpan cleaner | 11 | 0 | 100.00% | 12 | 0 | 100.00% | FALSE | 0.00% | 1.0000 |
|  | call box | 7 | 4 | 63.64% | 7 | 4 | 63.64% | FALSE | 0.00% | 1.0000 |
|  | toilet handle | 10 | 1 | 90.91% | 11 | 1 | 91.67% | TRUE | 0.76% | 1.0000 |
|  | room lightswitch | 10 | 1 | 90.91% | 11 | 1 | 91.67% | TRUE | 0.76% | 1.0000 |
|  | room chair | 6 | 5 | 54.55% | 7 | 5 | 58.33% | TRUE | 3.79% | 1.0000 |
|  | IV pole | 5 | 5 | 50.00% | 5 | 4 | 55.56% | TRUE | 5.56% | 1.0000 |
|  | bathroom lightswitch | 9 | 2 | 81.82% | 11 | 1 | 91.67% | TRUE | 9.85% | 0.5901 |
|  | bedside table | 6 | 5 | 54.55% | 8 | 4 | 66.67% | TRUE | 12.12% | 0.6802 |
|  | bathroom door closer | 8 | 3 | 72.73% | 11 | 1 | 91.67% | TRUE | 18.94% | 0.3168 |
|  | Total | 139 | 47 | 74.73% | 135 | 63 | 68.18% | FALSE | -6.55% | 0.1758 |
|  |  |  |  |  |  |  |  |  |  |  |
| MacConkey Agar | tray table | 10 | 1 | 90.91% | 7 | 4 | 63.64% | FALSE | -27.27% | 0.3108 |
|  | room sink | 10 | 1 | 90.91% | 9 | 3 | 75.00% | FALSE | -15.91% | 0.5901 |
|  | bedside table | 11 | 0 | 100.00% | 11 | 1 | 91.67% | FALSE | -8.33% | 1.0000 |
|  | bathroom sink | 9 | 2 | 81.82% | 9 | 3 | 75.00% | FALSE | -6.82% | 1.0000 |
|  | toilet rail | 8 | 3 | 72.73% | 8 | 4 | 66.67% | FALSE | -6.06% | 1.0000 |
|  | side rail | 11 | 0 | 100.00% | 11 | 0 | 100.00% | FALSE | 0.00% | 1.0000 |
|  | room door closer | 11 | 0 | 100.00% | 12 | 0 | 100.00% | FALSE | 0.00% | 1.0000 |
|  | bedpan cleaner | 11 | 0 | 100.00% | 12 | 0 | 100.00% | FALSE | 0.00% | 1.0000 |
|  | telephone | 10 | 1 | 90.91% | 11 | 1 | 91.67% | TRUE | 0.76% | 1.0000 |
|  | room chair | 10 | 1 | 90.91% | 11 | 1 | 91.67% | TRUE | 0.76% | 1.0000 |
|  | call box | 10 | 1 | 90.91% | 11 | 0 | 100.00% | TRUE | 9.09% | 1.0000 |
|  | toilet handle | 10 | 1 | 90.91% | 12 | 0 | 100.00% | TRUE | 9.09% | 0.4783 |
|  | room lightswitch | 10 | 1 | 90.91% | 12 | 0 | 100.00% | TRUE | 9.09% | 0.4783 |
|  | bathroom door closer | 10 | 1 | 90.91% | 12 | 0 | 100.00% | TRUE | 9.09% | 0.4783 |
|  | toilet seat | 9 | 2 | 81.82% | 11 | 1 | 91.67% | TRUE | 9.85% | 0.5901 |
|  | bathroom lightswitch | 9 | 2 | 81.82% | 11 | 1 | 91.67% | TRUE | 9.85% | 0.5901 |
|  | IV pole | 9 | 1 | 90.00% | 9 | 0 | 100.00% | TRUE | 10.00% | 1.0000 |
|  | Total | 168 | 18 | 90.32% | 179 | 19 | 90.40% | TRUE | 0.08% | 1.0000 |

**S1 Table. Contamination Frequency of Specific Surfaces Before & After the Intervention.** Surfaces are ordered by the change in the contamination frequency after the training intervention. P-values are for a two-tailed Fisher’s exact test. Counts from the full observation period are shown.
